# Experience of frontline nurses who managed the COVID-19 crisis: A qualitative study

**DOI:** 10.3389/fpubh.2023.1070916

**Published:** 2023-03-16

**Authors:** Seyed Tayeb Moradian, Hosein Mahmoudi

**Affiliations:** ^1^Atherosclerosis Research Center, Baqiyatallah University of Medical Sciences, Tehran, Iran; ^2^Trauma Research Center, Nursing Faculty, Baqiyatallah University of Medical Sciences, Tehran, Iran

**Keywords:** nursing, COVID-19, disaster planning [MeSH], coping skills, care burden

## Abstract

**Background:**

The health system was challenged during the COVID-19 pandemic. Nurses, as part of the health system, were expected to manage themselves in a situation where everyone was in crisis and to be able to do their work quietly and calmly. This study was conducted to show how Iranian nurses faced the COVID-19 crisis.

**Methods:**

In a qualitative content analysis study, 16 participants, including eight nurses, five supervisors, and three head nurses of a university hospital in Tehran, Iran, were interviewed between February and December 2020. Using purposive sampling, nurses who were working with patients with COVID-19 were selected to be involved. Data were analyzed using MAXQDA 10 software, and codes were categorized based on similarities and differences.

**Finding:**

Data analysis revealed 212 codes. These codes were classified based on similarities and differences in 16 categories, and four main themes emerged: unpreparedness, positive adaptation, negative coping, and reorganization.

**Conclusion:**

Since nurses are on the frontline in times of biological disaster, the COVID-19 pandemic provided an opportunity to demonstrate the role of nurses in reducing the burden of disease, identifying problems and opportunities, and planning appropriate interventions.

## Introduction

On 31 December 2019, Chinese health officials reported a cluster of cases of pneumonia of unknown cause (1). Later, the cause was identified as the novel coronavirus. It was later named COVID-19 and then SARS-CoV-2. After it spread to most countries, the World Health Organization (WHO) declared it an emergency and a pandemic (2, 3).

In the COVID-19 crisis, Iran was one of the most affected countries in the world. On 19 February 2020, the first case of COVID-19 was confirmed in Qom city, Iran. The disease then spread rapidly throughout the country (4).

This pandemic created many challenges for countries and their health systems. Increasing the number of deaths and involvement of all society sectors was a threat to global public health and the economy (5). After the severe acute respiratory syndrome (SARS) outbreak in 2003, hospitals, the nursing system, and nurses were expected to be well-prepared to deal with a similar viral epidemic (6). The year 2020 was named by the WHO as the Year of Nursing and Midwifery (7). Director-General Mr. Tedros stated that healthcare workers are “the glue that holds the health system and the outbreak response together” (8). Historically, nurses have played an important role in community health and the prevention and control of infection, but the COVID-19 situation was different (3).

Nurses faced an unprecedented challenge. On the one hand, they wanted to do their job properly, and on the other hand, personal protective equipment (PPE) was not sufficiently available (6). In addition to worrying about themselves, they feared contaminating relatives, friends, and other family members (9). Previous studies have shown that nurses experienced severe physical and psychological stress (10, 11). Every nurse has a story to tell, and they can help us to understand this situation better. This qualitative study was carried out to show how Iranian nurses faced the COVID-19 disaster.

## Methods

### Materials and methods

To understand how Iranian nurses faced the COVID-19 disaster, the conventional method of content analysis proposed by Graneheim and Lundman was used (12). In this study, using purposive sampling, 16 participants including eight nurses, five supervisors, and three head nurses working with COVID-19 patients were interviewed from February to December 2020. In this qualitative research, we did not have a predetermined sample size. Sampling continued until data saturation (13). This means that the exploration of participants' views and experiences was continued until the researchers concluded that there were no new data and the concepts were sufficiently captured.

Healthcare workers who were working with patients with COVID-19 and had extensive firsthand experience in caring for these patients were included. For enhancing the maximum variation, healthcare professionals, with different work experiences and different educational levels, were selected. Furthermore, the duration of working with patients with COVID-19 was a basis for sampling.

Data collection was performed using semi-structured interviews, each lasting from 20 to 45 min. Participants offered the interview place, usually in a quiet place in the hospital resting rooms.

Interviews were conducted by the first author of this article, an associate professor in nursing who has published several qualitative research papers and managed and supervised more than six Ph.D. projects using the qualitative research method. Some complementary interviews and data analysis were carried out by the second author, a professor in nursing who is experienced in emergency nursing and has good experience in qualitative research, especially the grounded theory method.

The initial questions of these interviews are the result of preliminary work in the field and the experiences and knowledge of the researchers. The interview guide was developed by the consensus of researchers. These questions were only a general framework to start the interviews, and later, with the progress of the interviews and the completion of the concepts, fundamental changes were made to the questions and their priority. The type and order of questions were changed as the data collection flow progressed. With the progress of the study and the formation of the categories, the subsequent interviews were guided to clarify the main categories and their connections.

Before each interview, the researcher introduced himself to build a good relationship and trust. Then, the objective of the research and the reason for interviewing were described. Next, written informed consent was obtained from the participants. After several warm-up questions, interviews began with a general open-ended question followed by more detailed and specific questions. Participants were asked to narrate their experiences in as much detail as possible. Probing and explanatory questions such as “Can you explain more?” Or “What did you mean by that?” were used for additional clarification of the answers given by participants. Finally, we asked the participants, “Is there any further data that you think will help us?” They were given the researcher's phone number and email address, and arrangements were made to contact them to confirm their statements or to have a follow-up interview. Two follow-up interviews were conducted for supplementary data and better explanations of quotations. Along with the interview text, participants' moods and characteristics, such as laughter and facial expressions, were also recorded.

The qualitative content analysis method was used for data analysis. Immediately after each interview, it was transcribed verbatim and then read several times to obtain a general sense of the participant's words. The MAXQDA 10 software was used for data analysis. First, meaning units were extracted, then they were coded and organized into different subcategories and categories based on their similarities and differences. Based on the interviews and constant comparison method, the underlying meanings were expressed in themes.

The Lincoln and Guba criteria for rigor and trustworthiness were used in this study (14). The researcher had a long-term engagement with the data and research field. Member checking was performed by summarizing the primary result of each interview and the final results with three nurses. In addition, the analysis process was carried out with the agreement of two members of the research team and was audited by two external supervisors. In cases of disagreement, discussion continued to reach an agreement. Furthermore, in the findings section, quotes from the participants are presented.

The aim and objectives of the study were clarified for participants and informed written consent was obtained from them. Moreover, their permission for recording the interviews was obtained. The time and place of the interviews were determined by participants and they were able to stop if they were exhausted or distressed.

### Findings

A total of 16 participants, including eight nurses, five supervisors, and three head nurses, were interviewed. The demographic characteristics are presented in Table 1. Data analysis revealed 212 codes, which were then classified based on similarities and differences into 15 categories. Further evaluation for hidden meaning resulted in the emergence of four main themes: unpreparedness, positive adaptation, negative coping, and reorganization. The details are presented in Table 2.

1. Unpreparedness: Based on the findings of this study, participants reported being surprised and unprepared as a major problem. This concept consists of four categories: surprise, shortage and deficiency, crowdedness, and inconsistency.
1-a. Surprise: Surprise was one of the main properties of this experience. For example, a supervisor with 26 years of experience said:

“*In the first Supervisor Shift after COVID-19, I realized that this is not a simple disease*.

*Standard accreditation programs and other programs were abruptly dropped. Even the ministry of health shut down auditing to improve hospitals' disaster management.”*

1-b. Shortage and deficiency: Another characteristic of this crisis was shortage and deficiency. An expert nurse with 16 years of experience said:

“*The first thing I remember at the beginning of the crisis was a severe shortage of equipment and human resources that was visible and remained until the following days. Emergency department nurses, as the frontline soldiers, sacrificed themselves to save the lives of patients, even without the effective PPE. Some of them later were infected.” [sic]*

1-c. Crowdedness: Crowding was a common experience for almost all participants. A nurse with 3 years of experience stated:

“*There were no longer empty beds, some patients were lying on the floor and tied their drugs to the curtain holders. When I went for dinner, more than 50 people were standing in line for fever and SPO*_2_ *check [sic].”*

1-d. Inconsistency: Disorganization and dissonance were other features of this crisis. For example, supervisors with 13 years of experience declared:

“*Of course, these inconsistencies existed until the last ward of the hospital was polluted by Corona.”*

Another supervisor with 26 years of experience said:

“*So the standards aren't useful at this time? Or…? Maybe saying these things in my language as a supervisor that trying to implement every single standard would be incorrect, but I still haven't answered my question.” [sic]*

2. Positive adaptation: Based on the findings of this study, participants used positive adaptation techniques, including self-sacrifice, prayer, spirituality, and improving patient mood.
2-a. Self-sacrifice: Sacrifice was one of the most seen behaviors during this crisis. A supervisor with 12 years of experience responded:

“*Thanks to God, despite the many problems, my colleagues using high responsibility and sacrificing themselves took great strides in controlling this great crisis.” [sic]*

2-b. Prayer and spirituality: Participants cited prayer as one of the positive methods of copping. A supervisor with 13 years of experience stated:

“*I wanted God to save us from this affliction. I have unconsciously remembered a verse from the Qur'an that says: In the stormy seas they read to me, and when they are saved, they don't remember who helped them to survive the storm. Of course, we are still in the stormy sea of Corona. We are praying the Seventh Prayer of Sajadiyya, but we read it in the hope of salvation from the storm of Corona, I hope we don't forget this exclamation after being saved from this storm. Remember these prayers that only God has saved us.”[sic]*

2-c. Improving patient mood: Another positive thing nurses did to adapt was to use the hospital's potential to improve patients' moods. The typical experiences of a nurse with 9 years of experience are described as follows:

“*The best thing was activating the hospital's advertising department to broadcast happy and exciting programs for patients through the hospital's internal network and the presence of clerics on [sic] patients' bedside during non-peak caring hours.”*

3. Reorganization: One of the positive ways to modify the existing organization system and make adjustments to manage the coronavirus was reorganization. This concept has multiple categories including making meeting the emergency needs, setting up a hospice, rationing, informing staff, and immediate encouragement of effective staff.
3-a. Meeting the emergency needs: emergency need to response the COVID-19 were managed in the organization. A supervisor with 14 years of experience said:

“*There were various suggestions for solving problems ranging from opening new departments to reducing emergency workload to using university nursing staff and senior nursing students to help. Almost all hospital wards were occupied by corona patients.”*

3-b. Setting up a hospice: Another positive way to deal with corona was to create a hospice to cut off the transmission chain, using public hospital facilities such as parking spaces to optimize space. A contributor said:

“*The important thing about the hospice is that it is a very interesting project, but it takes time to complete because it is a nascent plan.”*

3-c. Rationing: Managers followed a rationing strategy when faced with the threat of running out of equipment. A head nurse with 30 years of experience stated:

“*I rationed the staff with protective aids like masks, so we wouldn't get into trouble. I told the nurses and the other staff that the meal and pray program should be synchronized to reduce the consumption of masks and gloves.”*

3-d. Informing the staff: Nursing managers were tasked with explaining the standards of care to their colleagues. For example, a head nurse with 10 years of experience said:

“*I notified all the staff in the ward that the situation is not normal and that they have to comply more.” [sic]*

3-e. Immediate encouragement of effective staff: Another effective way of coping was to encourage and motivate nurses. A supervisor with 13 years of experience stated:

“*The incentive plans are needed to continue to work, today we had a good example. We hope this incentives continue, because they are imperative for maintaining the spirit.” [sic]*

4. Negative coping: Based on the findings of this study, some participants used negative coping mechanisms such as anxiety and fear of death, absence from work, sick leave, and feeling helpless in dealing with the crisis.
4-a. Anxiety and fear of death: The sense of anxiety and stress was common. This is how a nurse with 10 years of experience described his experience:

“*I get stressed out in the morning. I have a strange apprehension, but I have dedicated myself to God.”*

Another nurse with 12 years of experience said:

“*It was a bad night. Everyone's faces were angry. Nurses, physicians, and patients all had stress.”*

4-b. Sick leave: Some nurses, many of whom were women, used sick leave. A supervisor with 11 years of experience confirmed this, saying:

“*Some staff were not cooperating at this time; they used sick leave. They put pressure on other staff, especially those sacrificing themselves.”*

4-c. Helplessness: Due to the characteristics of the disease, some participants felt helpless and unable to deal with patients with COVID-19. A nurse with 15 years of experience said:

“*There was a time when you wanted to die from the severity of sadness, and it was a time when your friends were dying in front of your eyes and you couldn't do anything. It was really sad.”*

**Table 1:** Demographic characteristics of participants.

**Health care workers**		
Age, years (mean ± SD)	38.52 ± 5.60	
Gender	Male (%)	10 (62.5)
	Female (%)	6 (37.5)
Type of job	Supervisor (%)	3 (21.4)
	Head nurse (%)	5 (35.7)
	Nurse (%)	2 (14.3)
job experience, years (mean ± SD)	11 ± 4.72 (minimum 1 and maximum 22 years)	
Interview duration, minutes (mean ± SD)	34.58 ± 6.97	
Working with COVID-19 patients, months (mean ± SD)	6.12 ± 3.26 (minimum 1 and maximum 11 months)	

**Table 2:** Findings in the form of themes, sub-themes, and codes.

**Theme**	**Sub-theme**	**Code**
Unpreparedness	Surprising	Different from what we knew previously
	Shortage and deficiency	Lack of PPE
	Crowdedness	Unbearable number of patients
	Inconsistency	Working without standard
Positive adaptation	Prayer and spirituality	Reading the prayer
	improving patient mood	Celeries as mode improver
	Self-sacrifice	Putting oneself at risk to save the patient
Reorganization	Meeting the emergency needs	Opening a new ward to deal with crowding
	setting a hospice	A hospice to cut off the transmission chain
	Rationing	Rationing for managing shortages
	Informing the staff	Training on the standards of care
	Immediate encouragement of effective staff	Encouraging effective employees
Negative coping	Anxiety and fear of death	Feeling close to death
	Sick leave	Burden to others due to sick leave
	Helplessness	Severe sadness due to patient death

## Discussion

The study showed that hospitals and health systems were not prepared to deal with COVID-19. Although Iran was not the first country affected, and there was almost a preparedness in the country's officials, Iran's health systems were still surprised. One of the main concepts of the present study is a lack of preparedness. Experience has shown that, even after similar experiences, there is a vulnerability in health systems. The SARS crisis experience is similar to the COVID-19 pandemic, but the available trends show that insufficient lessons were learned from that crisis (15). During the flu outbreak of 1918, the actions of the affected countries were almost similar to the COVID-19 measures, for example, the quarantine measures (16). Studies show that tabletop maneuvering can be helpful, especially for countries that have not yet been affected by this type of crisis (17). The experiences of Iran, China, and other countries that have been affected by this problem can be helpful.

The lack of PPE equipment was another of the problems mentioned by nurses. Perhaps due to the awareness of the arrival of the disease, the equipment shortage in Iran was not severe, but this problem still existed. According to the interviews, part of this problem was due to differences in expectations. The nurses expected to wear the N95 mask and other protective clothing, but the managers wanted to comply with WHO and CDC recommendations, and these organizations did not recommend masks or other PPE to this extent. Of course, the recommendations changed over time.

A lack of PPE has also been reported in previous studies (18). Protection is one of the most important issues; when medical personnel are not properly protected, they will be infected. For example, approximately one-third of SARS victims were healthcare staff (19). There have also been reports of COVID-19 infections in healthcare workers (20). In other countries, the supply of surgical masks also declined and prices increased 5-fold (6).

In addition to fighting illness, nurses must risk their lives in difficult conditions such as a lack of protective equipment. In some hospitals in China, the staff was even forced to wear their raincoats and plastic bags for protection (19–21). Another key concept highlighted in this study is frequent management decisions for proper crisis management. The preparation of health centers plays an important role. Due to disaster-related shortages, most hospitals could not continue their routine for a week (22). Crisis resource depletion must be well-managed.

Another important category that emerged in this study is the psychological response to the crisis. Nurses experienced anxiety and fear of death. They were also concerned about contaminating family members. Psychological interventions for the treatment team were not well-planned.

Medical staff members were under severe physical and psychological pressure, and anxiety and stress were common (23). There were several causes of stress, from the lack of protective equipment to the fear of contaminating others (24, 25). In China, a series of psychological measures were planned and implemented. They also used online mental health services (26). In particular, interventions were needed for nurses. Nurses were expected to manage themselves in a situation where everyone was in crisis and to be able to do their work quietly and calmly (24).

Due to high psychological pressure, some staff members refused to return to work. Other studies also attribute this to the severity of psychological symptoms (27). For this reason, appropriate psychological interventions have been emphasized. Most nurses, of course, remained on the frontline to carry out their duties, and some of them died in the SARS 2002/2003 and COVID-19 crises while performing their duties (24).

The limitation of this study is related to the nature of the qualitative approach and the problem with generalizability. Most head nurses and supervisors in this hospital were men. As a result, in participant selection, most managers were men. This could result in gender bias.

## Conclusion

The results of this study show different aspects of nurses' responses to the interview questions, from negative to positive coping and adaptation. Lack of preparation and surprise were the initial observations and experiences of the healthcare system and nurses. Given that nurses are on the frontline, COVID-19 provided an opportunity to demonstrate the role of nurses in reducing the burden of illness, identifying problems and opportunities, and planning appropriate interventions. In addition, there is a large burden on the healthcare system and nurses, which should be considered in healthcare policymaking.

## Data availability statement

The raw data supporting the conclusions of this article will be made available by the authors, without undue reservation.

## Ethics statement

The studies involving human participants were reviewed and approved by Baqiyatallah University of Medical Sciences. The patients/participants provided their written informed consent to participate in this study. Written informed consent was obtained from the individual(s) for the publication of any potentially identifiable images or data included in this article.

## Author contributions

SM: study conception, design, and drafting of manuscript. HM and SM: acquisition, analysis, and interpretation of data. HM: critical revision. All authors contributed to the article and approved the submitted version.
